# Do orthopaedic surgeons agree on the willingness-to-randomize young patients with meniscal tears for repair versus resection? A video review study

**DOI:** 10.1016/j.ocarto.2026.100852

**Published:** 2026-07-28

**Authors:** B.A.M. Snoeker, R.W. Poolman, A. Turkiewicz, D.T. Grønne, S.A.F. Heijnen, H.A. Zuiderbaan, P. Neuman, D. Meuffels, M. Englund, J.B. Thorlund

**Affiliations:** a Clinical Epidemiology Unit, Orthopaedics, Department of Clinical Sciences Lund, Lund University, Lund, Sweden; b Department of Orthopaedics, University of Amsterdam, Amsterdam, the Netherlands; c Department of Orthopaedics, University of Leiden, Leiden, the Netherlands; d OLVG Hospital, Amsterdam, the Netherlands; e Center for Muscle and Joint Health, Department of Sports Science and Clinical Biomechanics, University of Southern Denmark, Odense, Denmark; f The Research and Implementation Unit PROgrez, Department of Physiotherapy and Occupational Therapy, Central and West Zealand Hospital, Slagelse, Denmark; g Bergman Clinics, Department of Orthopaedics, Breda, the Netherlands; h Velsen Clinics, Department of Orthopaedics, Velsen, the Netherlands; i Department of Orthopaedics, Skåne, University Hospital, Lund University, Malmö, Sweden; j Erasmus MC, Department of Orthopaedics, the Netherlands; k Research Unit for General Practice, Department of Public Health, University of Southern Denmark, Odense, Denmark

**Keywords:** Meniscal tears, Video review, Willingness-to-randomize, Feasibility study

## Abstract

**Objective:**

A randomized controlled trial (RCT) comparing meniscal repair versus resection is highly needed but may be difficult to perform due to strong surgeon treatment preferences. Therefore, we performed a pilot study to explore orthopaedic surgeons’ willingness-to-randomize meniscal tear patients to inform a future multicentre RCT.

**Method:**

Orthopaedic surgeons in The Netherlands (n = 26) and Sweden (n = 1) reviewed 19 arthroscopic video recordings of 19–46-year-old patients with isolated meniscal tears undergoing arthroscopic surgery (meniscal repair/resection). The orthopaedic surgeons reviewed the initial arthroscopic visualization of the knee joint and judged 1) whether they were willing-to-randomize the meniscal tear (yes/no), 2) which meniscal surgery they would perform on the tear (resection, repair, or no surgery), and 3) whether they preferred non-surgical treatment over surgical treatment (yes/no). We used Fleiss kappa with 95% confidence intervals (CI) to estimate agreement.

**Results:**

Twenty-seven knee surgeons with a median [range] of 12 [1–32] years of experience reviewed the videos. The Fleiss Kappa for agreement on whether to randomize a meniscal tear was 0.14 (95% CI 0.03–0.26). Agreement was 0.38 (95% CI 0.25–0.51) for the choice of meniscal surgery and 0.29 (95% CI 0.12–0.45) for preferred treatment option.

**Conclusion:**

Orthopaedic surgeons seem to exhibit little agreement as to whether they are willing to randomize a patient with an isolated meniscal tear, but also what treatment option they prefer. Our results suggest 1) an RCT for comparing resection vs repair is needed, and 2) alongside this RCT an observational experiment should evaluate if non-consensus results in pseudo-randomization.

## 1 Introduction

Meniscal tears in *young* (<40 years) patients may lead to functional disabilities in short-term, and increased risk of knee osteoarthritis (OA) in the long-term compared to persons without meniscal tears [1,2]. The choice of surgical treatment for meniscal tears is considered important to improve patient-reported pain and functional outcomes, and to potentially reduce the risk of people developing knee OA [3,4].

Arthroscopic partial meniscectomy (APM) is the most common orthopaedic procedure for treating meniscal tears worldwide, with several million procedures performed annually [3,5]. Nevertheless, current evidence suggests that meniscal repair may be the most favorable meniscal surgery in terms of restoring/preserving meniscal function, returning to sports, and protecting cartilage, rather than removing the damaged parts of the meniscus [6,7]. Most importantly, meniscal repair has been considered to provide better protection against the development of knee OA compared to APM [4,8,9]. Consequently, it has also been suggested that meniscal repair is more cost-effective than APM [10].

Despite these advantages, meniscal repair results in more re-interventions (∼25% in ACL intact knees for meniscal repair compared to ∼4% for APM up to 10 years after surgery), a higher complication rate, and less improvement in patient-reported outcomes [[11], [12], [13], [14]]. A significant limitation is that current evidence is based on observational studies with high risk of confounding by indication, i.e. risk of comparison of selected patients with different prognosis. No RCTs have compared APM vs repair for isolated meniscal tears in young patients [14]. However, performing a randomized controlled trial (RCT) is difficult due to strong treatment preferences among surgeons. One of the main arguments for surgeons not to randomize patients with a meniscal tear, is the amount of vascularization in the affected area. If the tear is in the vascularized red-red zone of the meniscus, orthopaedic surgeons are reluctant to perform APM, as the meniscus can potentially heal in this zone if treated with meniscal repair [15].

To inform a future multicentre RCT we aimed to explore orthopaedic surgeons’ willingness-to-randomize meniscal tear patients and their treatment preferences.

## 2 Methods

### 2.1 Design

In this feasibility study, we invited 36 orthopaedic surgeons in The Netherlands and one in Sweden to participate. Further, we recruited two additional orthopaedic surgeons to record 19 videos of patients with meniscal tears undergoing diagnostic arthroscopy, and subsequently (in the same surgery) either having meniscal repair or APM. Only anonymized videos of the diagnostic visualization of the joint and meniscus was shown to orthopaedic surgeons asked to review the videos. Orthopaedic surgeons signed informed consent before study participation. The study was approved by the Medical Ethics Committee in The Netherlands (2023.0690).

### 2.2 Videos

Arthroscopic videos of knees in patients aged 19–46 years with an isolated meniscal tear were recorded in 2023. Videos of patients were excluded if the patient had I) fractures of the affected extremity within the previous 6 months, II) had previous knee surgery on the affected knee, or III) had a comorbidity requiring treatment other than meniscal tears (e.g. ACL injury).

The meniscus videos were collected from two orthopaedic centers (one public hospital and one private clinic in the Netherlands). The videos were anonymized and then saved on an online platform that was accessible only with a password. Orthopaedic surgeons were eligible to rate the videos if they had a minimum of one year of experience as an orthopaedic knee surgeon. Orthopaedic surgeons participating in the study received access to the platform to watch the videos. Participating surgeons were aware of the in- and exclusion criteria but did not receive additional clinical information. For each video, the orthopaedic surgeons judged 1) whether they were willing-to-randomize the patient with the meniscal tear shown on the video (yes/no), 2) which meniscal surgery they would perform on that meniscal tear (resection, repair, or no surgery), and 3) whether they preferred conservative (non-surgical) treatment over surgical treatment for this type of meniscal tear (yes/no). They recorded their findings in an Excel file. The surgeons rated the videos independently and were not aware of their colleagues’ responses nor of which treatment the patient actually received. One orthopaedic surgeon reported the type of meniscal tear, its location, and the vascularization of the area based on the Cooper classification, that takes the vascularization of the different meniscus sections into account [16].

### 2.3 Statistical analysis

We based our sample size on the following calculations. We generated up to 600 simulated datasets of artificial data for 18 different scenarios, assuming prevalence of answer “yes” from almost 0 to almost 1 and varying degrees of agreement between the raters. After running all scenarios, we concluded that we needed 30 raters (orthopaedic surgeons) and 20 patients’ videos to estimate an agreement coefficient with acceptable precision (width of 95% confidence intervals (CI) for kappa at most around 0.4).

Our primary outcome was the willingness of orthopaedic surgeons to randomize patients with meniscal tears to either resection or repair. The secondary outcomes were the preference for the type of meniscal surgery to perform, and whether non-surgical treatment was preferred over meniscal surgery. If a video review was missing, we still analyzed all available data from that reviewer. We analyzed agreement using Fleiss Kappa. Fleiss Kappa can be interpreted as the more well-known Cohen's Kappa, where values greater than 0.75 represent excellent agreement, values between 0.40 and 0.75 represent fair to moderate agreement, and values below 0.40 represent poor agreement. We reported the Fleiss Kappa coefficients with their 95% CI.

## 3 Results

### 3.1 Descriptives

The meniscus videos contained 19 patients aged 19–46 years (32% female). Nine patients (47%) had a medial meniscal tear (Table 1). There were 12 posterior meniscal tears, 6 posterior-body tears, and one tear located at the body. There were no anteriorly located meniscal tears. The location and type of each individual meniscal tear are presented in Table 2.

**Table 1:** Baseline characteristics of meniscal tear patients.

Characteristic	Baseline value
Age, median (range)	31 (19/46)
Sex, female, n (%)	6 (32)
Left knee, n (%)	7 (37)
Type of surgery performed	
*APM, n (%)*	9 (47)
*Meniscal repair, n (%)*	6 (32)
*APM and meniscal repair, n (%)*	4 (21)
Meniscal tear type	
*Bucket handle, n (%)*	3 (17)
*Bucket handle complex, n (%)*	1 (5)
*Complex: longitudinal + horizontal + flap, n (%)*	1 (5)
*Degenerative with flaps, n (%)*	1 (5)
*Degenerative complex, n (%)*	1 (5)
*Flap, n (%)*	5 (26)
*Horizontal, n (%)*	1 (5)
*Horizontal complex, n (%)*	1 (5)
*Longitudinal, n (%)*	3 (17)
*Longitudinal with flap, n (%)*	1 (5)
*Radial, n (%)*	1 (5)
Location of meniscal tear, medial, n (%)	9 (47)

**Table 2:** Location and type of meniscal tears per video.

Case #	Meniscal tear type	Medial/lateral meniscal tear	Location of meniscal tear	Cooper classification	Meniscal surgery performed
1	Complex: longitudinal + horizontal + flap	Lateral	Posterior	F2-3	APM
2	Flap	Medial	Posterior	A2-3	APM
3	Bucket-handle	Lateral	Body-posterior	EF1	APM
4	Longitudinal	Lateral	Posterior	F2	APM
5	Bucket-handle	Lateral	Posterior	F0-1	Meniscal repair
6	Horizontal	Lateral	Posterior	F1-3	Meniscal repair and APM
7	Bucket-handle complex	Medial	Body-posterior	AB1	Meniscal repair
8	Horizontal complex	Lateral	Body-posterior	EF2-3	Meniscal repair and APM
9	Longitudinal	Medial	Posterior	A0-1	APM
10	Flap	Medial	Body-posterior	AB2-3	APM
11	Degenerative with flaps	Medial	Posterior	A3	APM
12	Bucket-handle	Lateral	Posterior	F1	APM
13	Flap	Medial	Body	B3	Meniscal repair
14	Degenerative complex	Medial	Posterior	A3	APM
15	Flap	Lateral	Posterior	F3	Meniscal repair
16	Radial	Lateral	Body-posterior	EF2-3	Meniscal repair
17	Flap	Medial	Posterior	A2-3	Meniscal repair and APM
18	Longitudinal	Medial	Posterior	A0-1	Meniscal repair and APM
19	Longitudinal with flap	Lateral	Body-posterior	EF2-3	Meniscal repair

In total, 27 orthopaedic knee surgeons from 17 different institutions (general hospitals, academic institutes, and private clinics) in The Netherlands and from one hospital in Sweden reviewed the meniscus videos. They had a median of 12 years of experience (range 1–32 years). Two surgeons only rated 18 meniscus videos, and another orthopaedic surgeon only rated 17 of the 19 meniscus videos as they felt unable to judge the meniscal tear shown. We observed much variation in the willingness-to-randomize between orthopaedic surgeons. For some meniscal tears, the choice of meniscal surgery was fairly consistent among surgeons. Results of the ratings for all three outcomes are shown in Table 3.

**Table 3:** Rating of the meniscal tears by orthopaedic surgeons for willingness-to-randomize, choice of meniscal surgery and preferred treatment option.

	Willingness-to-randomize		Choice of meniscal surgery			Preferred treatment: Conservative	
Case #	*Yes*	*No*	*APM*	*Repair*	*No surgery*	*Yes*	*No*
1	15	12	18	5	4	14	13
2	4	23	26	1	0	4	23
3	16	11	5	22	0	2	25
4	21	6	4	20	3	7	20
5	7	19	0	26	0	0	26
6	24	3	8	10	9	22	5
7	15	12	8	19	0	0	27
8	22	5	17	5	5	15	12
9	11	16	0	24	3	9	18
10	11	15	24	2	0	9	17
11	10	17	14	2	11	23	4
12	24	3	3	22	2	8	19
13	7	19	16	1	9	23	3
14	12	15	18	6	3	19	8
15	19	8	2	21	4	13	14
16	16	10	10	16	0	6	20
17	15	12	20	1	6	21	6
18	12	15	0	25	2	10	17
19	19	8	22	3	2	16	11

### 3.2 Agreement

The Fleiss Kappa for agreement on whether to randomize a meniscal tear was 0.14 (95% CI 0.03–0.26). Agreement for the choice of meniscal surgery (resection/repair/no surgery) was 0.38 (95% CI 0.25–0.51), and for preferred treatment option (conservative treatment vs surgery) 0.29 (95% CI 0.12–0.45).

**Fig. 1:**
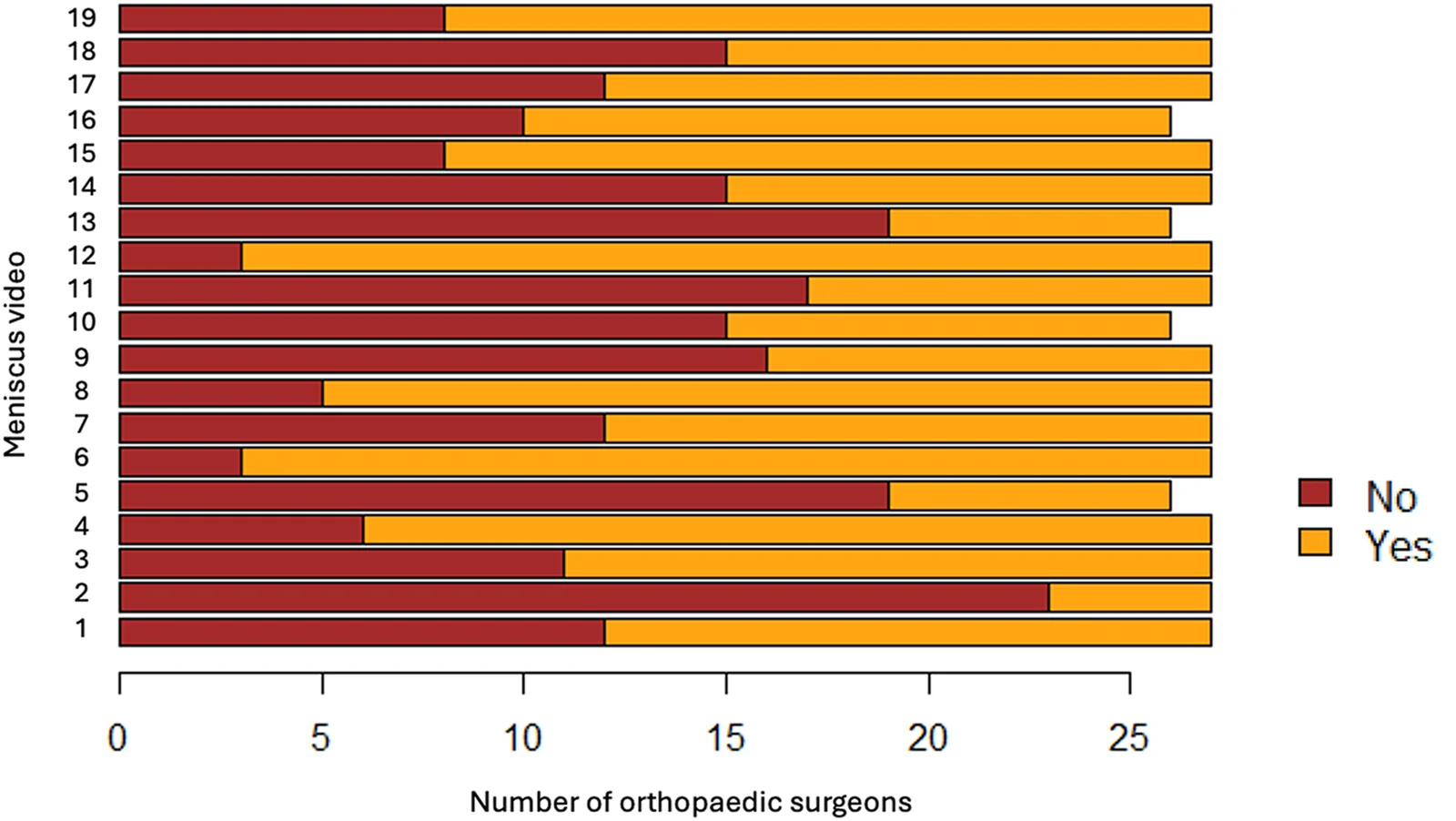
Willingness-to-randomize meniscal tears by orthopaedic surgeons based on the meniscus videos.

**Fig. 2:**
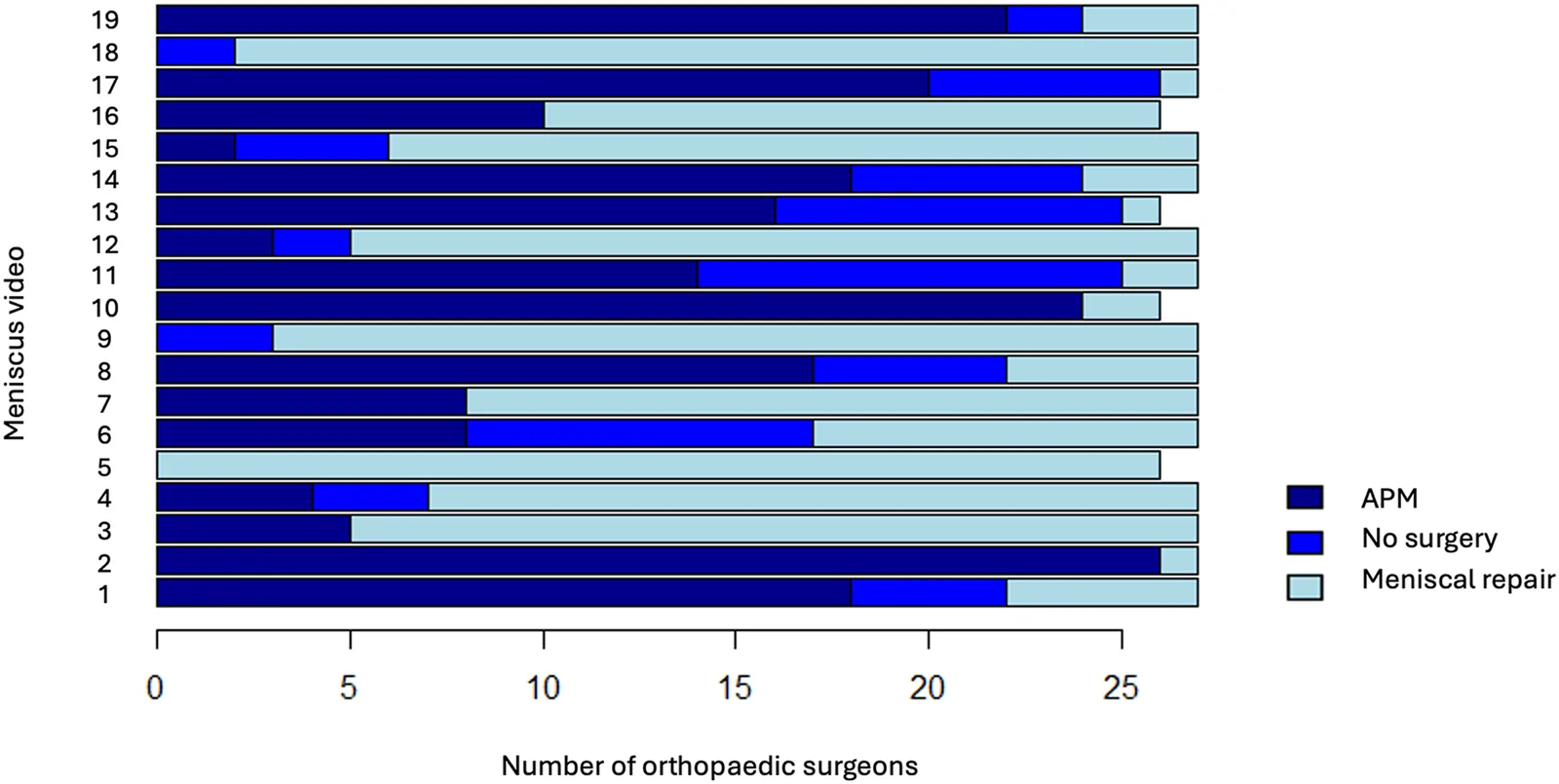
The distribution of the choice of treatment for the 19 included cases.

The kappa coefficients and their CI indicate that the majority of meniscal tears were not consistently rated, in line with observed high variability in judgment between surgeons. The only exception was for meniscal tear cases #6 and #12, where almost all surgeons (n = 24) were willing to randomize the meniscal tear on the video (Fig. 1, Table A1 in Appendix). Although both are lateral posterior meniscal tears in a similar Cooper zone (F1), the performed surgery was for case #6 meniscal repair and APM, while case #12 only had APM. Also, the choice of treatment (APM, meniscal repair or no surgery) for these specific videos varied much across the surgeons (Fig. 2).

Although we observed considerable variation between the surgeons in the choice of meniscal surgery, we also noticed that for cases # 5, 9, and 18 the surgeons generally agreed on performing a meniscal repair (Fig. 2). The tears from cases #9 and #18 were very similar, with a longitudinal medial posterior meniscal tear in the vascularized area (red-red zone). Also, the meniscal tear from case #5 was in the red-red zone, although it was a lateral posterior bucket-handle tear. These meniscal tears were surgically treated differently; case #5 had meniscal repair, case #9 had APM, and case #18 had both meniscal repair and APM.

Except for two cases, at least a few orthopaedic surgeons preferred non-surgical treatment over a meniscal surgery based on the meniscal tear shown on the video.

## 4 Discussion

We found poor agreement in the willingness-to-randomize young patients with a meniscal tear to APM or meniscal repair. However, the majority of meniscal tears were graded as suitable for randomization by at least 30% of surgeons. Poor agreement was also observed with respect to which meniscal surgery should be performed, or if treatment should be surgical or non-surgical. The poor agreement suggests poor consensus on the preferred treatment of meniscal tears in young patients with isolated meniscal tear supporting the need for an RCT. It further suggests that the majority of isolated meniscal tears in young patients could be suitable for randomization.

Meniscal repair has gained popularity as it is believed to reduce the risk of knee OA in the long term, in contrast to APM which is associated with increased risk for knee OA [4,6,7,9,17]. However, all existing studies comparing meniscal repair and APM are observational studies with a high risk of confounding by indication, which can lead to a comparison of selected groups of patients with different prognoses. An RCT provides stronger support for which surgical procedure to prefer in young patients with isolated meniscal tears that are considered eligible for surgery.

Performing an RCT is challenging, due to orthopaedics surgeons’ preference for one or the other treatment option for each specific patient. Although most surgeons participating in our study considered the treatment choice (i.e. repair or APM) for a young patient with a meniscal tear to be clear, our study revealed that there was little consistency among which *meniscal surgery* should be performed. Importantly, there was also low agreement on which tear was suitable for *randomization*, suggesting that most isolated tears in young patients could potentially be randomized.

For most of the case videos, the surgeons did not agree whether they were willing-to-randomize the meniscal tear shown. Only for two case videos 24/27 raters agreed on the appropriateness to randomize. The videos contained two different types of meniscal tears (radial or longitudinal tears), and the patients underwent two different procedures (APM and meniscal repair, not known by the rating surgeons). Because there was only one radial tear, we cannot conclude that the agreement was based on this meniscal tear type. However, there were multiple longitudinal meniscal tears, and from our data we can confirm that orthopaedic surgeons did not agree on the willingness-to-randomize for all longitudinal meniscal tears.

Another interesting finding from these two case videos, #6 and #12, is that both tears were in a vascularized area, which was one of the main arguments for the surgeons not to be willing to randomize a meniscal tear. Nevertheless, most observers (24/27) were willing to randomize these meniscal tears.

For meniscal surgery, we found that for some of the case videos, orthopaedic surgeons were in favor of a certain type of meniscal surgery. For example, for case video #5, all surgeons selected meniscal repair. Despite this strong preference, 7 out of 26 surgeons were still willing to randomize to provide evidence on whether this was in fact the case. We see similar results in case videos #9, 12, and 18 for meniscal repair, and in case video #10 for APM. Although 24 out of 26 orthopaedic surgeons prefer APM in the case #10, 11 surgeons were willing-to-randomize this patient either to meniscal repair or APM. Thus, even if for some menisci there is large agreement on which type of meniscal surgery should be performed, there is a relatively poor agreement in the willingness-to-randomize. Also, in situations where 80% of the surgeons have a preferred surgery (either APM or meniscal repair), more than half of the surgeons is willing to randomize, implicating that there is still uncertainty about what is the optimal surgery. The choice for surgery type did not seem to depend on the years of experience as a knee surgeon.

We are aware that a wide range of meniscal tears have been included. The meniscal tears included reflect the criteria in the actual planned RCT and the real-world situation. Given the variability in responses across all included tear types, there is no evidence that our inclusion criteria explain the lack of agreement. Rather, it highlights that the decision whether a person is supposed to be randomized, should not be based on the surgeon's preferences, but rather strictly based on the inclusion criteria of the trial.

We also asked whether non-surgical treatment was preferred over surgery. It was a challenging question, as the rating surgeon was aware that the patient had already begun an arthroscopy. Nevertheless, there were still quite some raters who preferred a non-surgical treatment for tears that were operated on, although agreement was poor. It could be due to a general shift towards non-surgical management before opting for surgery, since a trial found no essential differences between the two treatment strategies [18].

Lastly, an interesting finding is that there is only minor overlap between the answer “no surgery” to the question of which meniscal surgery they would perform on that video case and “conservative” to the question whether they preferred non-surgical treatment over surgical treatment. It is thus unclear for which other treatment raters could have opted.

Our results suggest that clinical equipoise is present – meaning that there is little agreement on which surgery to prefer for which meniscal tear, which provides a strong argument for an RCT. Given the poor inter-rater agreement, the non-consensus can result in pseudo-randomization, if applied in a setting with common patient selection and inclusion/exclusion criteria, making causal inference feasible. Consequently, we suggest evaluating a “natural experiment” alongside any future RCT for orthopaedic surgeons who are not willing to randomize patients into one of the surgical approaches. The surgeons decide before the start of the study whether they are willing to randomize patients, or whether they are only comfortable by their own judgement. In this pilot study we observed poor consensus between surgeons in the choice for type of meniscal surgery, thus we consider the choice of treatment to resemble random assignment.

Only a few observational studies included patients between 18 and 40 years with an isolated meniscal tear, so the proposed experiment can provide valuable new insights [14,17]. The concept is innovative because it utilizes the natural variance in clinical judgment when applied in a well-defined clinical study to create a randomized effect in a non-randomized study setting, thereby enabling causal inferences that are not attainable in traditional observational studies where no natural experiment occurs. It also reflects real/world variability as it allows for inclusion of surgeons and patients who are not willing to participate in an RCT and is cost-effective. It's a creative adaptation to the challenges faced in surgical research. It could serve as a model for other areas where RCTs are challenging to implement due to strong practitioner preferences or other constraints.

Our study has some limitations. The case videos were short, and the surgeons were unaware of the clinical information of the patients while rating the videos. Only the in- and exclusion criteria of this study were known. Clinical information is an important part of the clinical decision-making process, and therefore we acknowledge that the information provided to surgeons to make a decision was not identical to usual clinical practice. However, we do believe for our primary study purpose, to determine whether an orthopaedic surgeon is willing to randomize a meniscal tear, the information was sufficient. One of the main arguments not to randomize a meniscal tear was the location of the tear (vascularized/non-vascularized area), which was clearly visible on the videos. There were 7 meniscal tears in the red-red zone, 8 in the red-white zone, and 4 in the white-white zone, making it plausible that vascularization of the meniscal tear was a factor influencing the willingness to randomize. Another limitation was the quality of the videos. Although the visibility was acceptable, it was difficult to obtain the full picture of the entire meniscus. One strength of our study is that we included 27 knee surgeons from different types of institutions (general hospitals, academic institutes, and private clinics), and showed them almost 20 different meniscus videos. Furthermore, our CI on our estimates were narrow with a clear interpretation of poor agreement.

## 5 Conclusions

Orthopaedic surgeons seem to exhibit little agreement as to whether they are willing to randomize an isolated meniscal tear, but also what treatment option is best on 18–40-year-old patients with isolated meniscal tears. These findings justify a well-designed RCT for comparing resection vs repair. It also highlights opportunities for natural experimental evaluation alongside this RCT if non-consensus results in pseudo-randomization, making causal inference feasible.

## Author contributions

1) Conception and design of the study, or acquisition of data, or analysis and interpretation of data
Barbara Snoeker, Jonas Bloch Thorlund, Martin Englund, Aleksandra Turkiewicz, and Rudolf Poolman were involved in the conception and design of the study, and obtained funding. Duncan Meuffels, Paul Neuman, Dorte Grønne, Stefan Heijnen and Aernout Zuijderbaan were involved in the provision of study materials or patients, the collection and assembly of the data, and administrative, technical and logistic support. Analysis of the data has been performed by Barbara Snoeker and Aleksandra Turkiewicz, all authors were involved by the interpretation of the data. Aleksandra Turkiewicz was the statistical expert.
(2) drafting the article or revising it critically for important intellectual content
Barbara Snoeker, Jonas Bloch Thorlund, Martin Englund, Aleksandra Turkiewicz, and Rudolf Poolman were involved in drafting the article. All authors critically revised the article for important intellectual content.
(3) final approval of the version to be submitted

All authors approved the final version of the manuscript for submission.

Barbara Snoeker, Jonas Bloch Thorlund, Martin Englund, Aleksandra Turkiewicz, and Rudolf Poolman take responsibility for the integrity of the work from inception to finished article.

## Role of the funding source

This study has been funded by Greta and Johan Kock Foundations, The 10.13039/501100004359Swedish Research Council, The 10.13039/501100007949Swedish Rheumatism Association, and The Foundation for People with Movement Disability in Skåne.

## Conflicts of interest

AT is associate editor for statistics at Osteoarthritis and Cartilage. ME reports consultancy for Grünenthal Sweden AB and Genascence.

## Figures and Tables

**Table A1:** Orthopaedic surgeon's willingness to randomize per meniscusvideo

	Orthopaedic surgeons willing to randomize (yes/no)																										
Videos	**1**	**2**	**3**	**4**	**5**	**6**	**7**	**8**	**9**	**10**	**11**	**12**	**13**	**14**	**15**	**16**	**17**	**18**	**19**	**20**	**21**	**22**	**23**	**24**	**25**	**26**	**27**
1	Yes	No	No	Yes	Yes	Yes	No	Yes	Yes	Yes	No	No	Yes	No	Yes	No	Yes	Yes	No	No	Yes	Yes	Yes	No	Yes	No	No
2	No	No	No	No	No	No	No	Yes	No	No	No	No	No	No	Yes	Yes	No	No	No	No	No	No	Yes	No	No	No	No
3	Yes	Yes	No	No	No	Yes	Yes	No	No	No	Yes	Yes	Yes	Yes	Yes	No	Yes	Yes	Yes	No	No	Yes	No	Yes	Yes	Yes	No
4	Yes	Yes	No	No	No	Yes	Yes	Yes	Yes	Yes	Yes	Yes	No	Yes	Yes	Yes	Yes	Yes	Yes	Yes	Yes	No	No	Yes	Yes	Yes	Yes
5	No	Yes	No	No	No	No	No	No	No	No	Yes	Yes	No	No	Yes	No	Yes	No	No	Yes	No	/	No	No	No	No	Yes
6	Yes	Yes	Yes	Yes	Yes	Yes	Yes	Yes	Yes	Yes	Yes	No	Yes	Yes	Yes	Yes	Yes	Yes	No	Yes	Yes	Yes	Yes	Yes	Yes	Yes	No
7	No	No	No	Yes	No	No	Yes	No	Yes	Yes	Yes	Yes	Yes	No	Yes	No	Yes	No	No	No	No	Yes	Yes	Yes	Yes	Yes	Yes
8	Yes	No	Yes	Yes	Yes	Yes	Yes	Yes	Yes	Yes	Yes	No	Yes	Yes	Yes	Yes	Yes	Yes	No	Yes	Yes	Yes	Yes	No	Yes	Yes	No
9	No	Yes	No	No	No	Yes	No	No	Yes	No	Yes	Yes	No	No	Yes	No	No	Yes	Yes	Yes	No	No	No	Yes	No	No	Yes
10	No	No	No	No	Yes	No	No	/	No	Yes	No	Yes	No	Yes	Yes	Yes	Yes	Yes	No	Yes	Yes	No	Yes	No	No	No	No
11	No	No	No	No	No	No	No	Yes	No	No	Yes	Yes	No	Yes	Yes	No	Yes	Yes	No	Yes	No	No	No	No	Yes	Yes	No
12	Yes	Yes	Yes	Yes	Yes	Yes	Yes	Yes	Yes	Yes	Yes	Yes	Yes	No	Yes	Yes	Yes	Yes	Yes	Yes	No	Yes	No	Yes	Yes	Yes	Yes
13	No	No	No	No	No	No	No	/	No	Yes	Yes	No	No	Yes	Yes	No	No	Yes	No	Yes	Yes	No	No	No	No	No	No
14	No	No	No	No	No	No	No	Yes	Yes	Yes	No	No	Yes	Yes	Yes	No	No	Yes	No	Yes	Yes	No	Yes	Yes	No	Yes	No
15	No	Yes	No	Yes	Yes	Yes	No	No	Yes	Yes	Yes	Yes	No	Yes	Yes	No	Yes	Yes	Yes	Yes	Yes	No	No	Yes	Yes	Yes	Yes
16	Yes	No	No	Yes	No	No	Yes	No	Yes	Yes	Yes	Yes	Yes	Yes	Yes	Yes	Yes	Yes	No	No	No	Yes	Yes	No	No	Yes	/
17	No	No	No	Yes	No	No	No	Yes	Yes	Yes	Yes	No	Yes	Yes	Yes	Yes	No	Yes	No	Yes	Yes	No	No	Yes	Yes	Yes	No
18	No	Yes	No	No	No	Yes	No	No	No	No	Yes	Yes	No	Yes	Yes	No	Yes	Yes	Yes	Yes	No	No	No	Yes	No	No	Yes
19	Yes	No	Yes	Yes	Yes	Yes	No	Yes	No	Yes	Yes	No	Yes	Yes	Yes	Yes	No	Yes	No	Yes	Yes	Yes	Yes	Yes	Yes	No	No

## References

[1] Poulsen E., Goncalves G.H., Bricca A., Roos E.M., Thorlund J.B., Juhl C.B. (2019). Knee osteoarthritis risk is increased 4-6 fold after knee injury – a systematic review and meta-analysis. Br. J. Sports Med..

[2] Snoeker B., Turkiewicz A., Magnusson K., Frobell R., Yu D., Peat G. (December 2019). Risk of knee osteoarthritis after different types of knee injuries in young adults: a population-based cohort study. Br. J. Sports Med..

[3] Khan M., Evaniew N., Bedi A., Ayeni O.R., Bhandari M. (2014). Arthroscopic surgery for degenerative tears of the meniscus: a systematic review and meta-analysis. CMAJ.

[4] Persson F., Turkiewicz A., Bergkvist D., Neuman P., Englund M. (2018 Feb). The risk of symptomatic knee osteoarthritis after arthroscopic meniscus repair vs partial meniscectomy vs the general population. Osteoarthr. Cartil..

[5] Abram S.G.F., Judge A., Beard D.J., Wilson H.A., Price A.J. (2019). Temporal trends and regional variation in the rate of arthroscopic knee surgery in England: analysis of over 1.7 million procedures between 1997 and 2017. Has practice changed in response to new evidence?. Br. J. Sports Med..

[6] Rongen J.J., Rovers M.M., van Tienen T.G., Buma P., Hannink G. (2017). Increased risk for knee replacement surgery after arthroscopic surgery for degenerative meniscal tears: a multi-center longitudinal observational study using data from the osteoarthritis initiative. Osteoarthr. Cartil..

[7] Roemer F.W., Kwoh C.K., Hannon M.J., Hunter D.J., Eckstein F., Grago J. (2017). Partial meniscectomy is associated with increased risk of incident radiographic osteoarthritis and worsening cartilage damage in the following year. Eur. Radiol..

[8] Hurmuz M., Ionac M., Hogea B., Miu C.A., Tatu F. (2024 Mar 30). Osteoarthritis development following meniscectomy vs. meniscal repair for posterior medial meniscus injuries: a systematic review. Medicina (Kaunas).

[9] Migliorini F., Schäfer L., Bell A., Weber C.D., Vecchio G., Maffulli N. (2023 Dec). Meniscectomy is associated with a higher rate of osteoarthritis compared to meniscal repair following acute tears: a meta-analysis. Knee Surg Sports Traumatol Arthrosc.

[10] Rogers M., Dart S., Odum S., Fleischli J. (2019 Dec). A cost-effectiveness analysis of isolated meniscal repair versus partial meniscectomy for red-red Zone, vertical meniscal tears in the young adult. Arthroscopy.

[11] Boric-Persson F., Turkiewicz A., Englund M., Neuman P. (2025 Jul). Increased reoperation rates after meniscus repair compared to arthroscopic partial meniscectomy: data from a comprehensive clinical cohort with up to 10 years follow-up. Knee Surg. Sports Traumatol. Arthrosc..

[12] Paxton E.S., Stock M.V., Brophy R.H. (2011). Meniscal repair versus partial meniscectomy: a systematic review comparing reoperation rates and clinical outcomes. Arthroscopy.

[13] Nepple J.J., Block A.M., Eisenberg M.T., Palumbo N.E., Wright R.W. (2022 Jul 20). Meniscal repair outcomes at greater than 5 years: a systematic review and meta-analysis. J Bone Joint Surg Am.

[14] Pihl K., Englund M., Christensen R., Lohmander L.S., Jørgensen U., Viberg B. (2021). Less improvement following meniscal repair compared with arthroscopic partial meniscectomy: a prospective cohort study of patient-reported outcomes in 150 young adults at 1-and 5-years follow-up. Acta Orthop..

[15] Beaufils P., Pujol N. (2017 Dec). Management of traumatic meniscal tear and degenerative meniscal lesions. Save the meniscus. Orthop Traumatol Surg Res.

[16] Cooper D.E., Arnoczky S.P., Warren R.F. (1990 Jul). Arthroscopic meniscal repair. Clin. Sports Med..

[17] Stein T., Mehling A.P., Welsch F., von Eisenhart-Rothe R., Jäger A. (2010 Aug). Long-term outcome after arthroscopic meniscal repair versus arthroscopic partial meniscectomy for traumatic meniscal tears. Am. J. Sports Med..

[18] Skou S.T., Hölmich P., Lind M., Jensen H.P., Jensen C., Garval M. (2022). Early surgery or exercise and education for meniscal tears in young adults. NEJM Evid..

